# Ultrafast Modulation of THz Waves Based on MoTe_2_-Covered Metasurface

**DOI:** 10.3390/s23031174

**Published:** 2023-01-19

**Authors:** Xing Xu, Jing Lou, Mingxin Gao, Shiyou Wu, Guangyou Fang, Yindong Huang

**Affiliations:** 1 Aerospace Information Research Institute, Chinese Academy of Sciences, Beijing 100094, China; 2 Innovation Laboratory of Terahertz Biophysics, National Innovation Institute of Defense Technology, Beijing 100071, China; 3 Key Laboratory of Electromagnetic Radiation and Sensing Technology, Chinese Academy of Sciences, Beijing 100190, China; 4 School of Electronic, Electrical and Communication Engineering, University of Chinese Academy of Sciences, Beijing 100049, China

**Keywords:** photo-switching, terahertz, reconfigurable metasurface, ultrafast dynamics

## Abstract

The sixth generation (6G) communication will use the terahertz (THz) frequency band, which requires flexible regulation of THz waves. For the conventional metallic metasurface, its electromagnetic properties are hard to be changed once after being fabricated. To enrich the modulation of THz waves, we report an all-optically controlled reconfigurable electromagnetically induced transparency (EIT) effect in the hybrid metasurface integrated with a 10-nm thick MoTe_2_ film. The experimental results demonstrate that under the excitation of the 800 nm femtosecond laser pulse with pump fluence of 3200 μJ/cm^2^, the modulation depth of THz transmission amplitude at the EIT window can reach 77%. Moreover, a group delay variation up to 4.6 ps is observed to indicate an actively tunable slow light behavior. The suppression and recovery of the EIT resonance can be accomplished within sub-nanoseconds, enabling an ultrafast THz photo-switching and providing a promising candidate for the on-chip devices of the upcoming 6G communication.

## 1. Introduction

Located in the electromagnetic spectrum between the microwave and infrared region, the terahertz (THz) frequency band is a spectral window with great scientific interests [1,2,3]. Nowadays, the THz technology has found wide applications, including nondestructive sensing [4], biomedicine [5], security inspection [6], and communication [7,8,9]. As one of the most promising applications, the THz wireless communication has attracted tremendous attention in promoting the development of the sixth generation (6G) communication network, and is expected to enable the implementation of the “internet of everything” in the near future [10,11,12]. Generally, THz radiation can offer higher carrier frequency and spatial resolution than the microwave does. Meanwhile, it can penetrate a large number of the non-polar non-metallic substances, such as the silicon, plastic, clothing and paper, opening the way of novel communication to be used by satellites, autonomous cars, smart cities and so on [13,14,15]. It requires a series of regulations to THz waves when using as the 6G communication carriers. However, the limited availability of THz materials in nature prevents the flexible modulation of THz radiation [16].

The appearance of metasurfaces has provided an effective solution to modulate THz waves [17,18,19]. By altering the geometry and arrangement pattern of sub-wavelength meta-atoms in metasurfaces, the amplitude, phase and polarization of THz waves can be engineered, presenting numerous unusual applications, such as ultrathin flat lenses [20,21], THz broadband filter [22], and THz vortex beam generation [23,24]. Generally, once a planar metal structure is fabricated, its electromagnetic (EM) properties are unable to be actively tuned, which blocks the further flexible regulation of EM waves. To solve this problem, the concept of reconfigurable metasurfaces comprised of the active media and meta-atoms has been proposed [25,26,27]. By introducing the external excitation such as the optical [28,29,30,31,32,33], electrical [34,35,36], thermal [37,38,39] and mechanical stimuli [40,41], the intrinsic properties or physical form of the active media can be changed, affecting the EM properties of the metasurface. For example, for the THz asymmetric split ring resonators (TASRs) covered by a 310-nm thick germanium (Ge) film, photogenerated carriers can be excited by the 800 nm pump laser stimulus to change the resonance state [32]. Specifically, without pump laser excitation, the TASRs exhibits Fano resonance for the incident polarized THz radiation, denoted as the “on” state. When the pump laser reaches a threshold energy, the Fano resonance can be quenched, denoted as the “off” state. Moreover, compared with the electrical, thermal, and mechanical stimuli, the optical excitation possesses the unique advantage of ultrafast response speed. This means that the switch between “on” and “off” states can be achieved within picoseconds to nanoseconds. It shows the potential of providing an ultrafast data processing speed that is applicable in the 6G communication.

To realize the ultrafast switchable resonance state, one key point is to utilize the active semiconductor materials. Photocarrier dynamics in the active materials directly affect the switching time and modulation depth of the resonance. Nowadays, two-dimensional transition metal dichalcogenides (TMDCs) have received much attention due to their excellent photoelectric properties, such as the adjustable bandgap with the layer, high carrier mobility and good stability in the atmosphere [42,43]. MoTe_2_ is a significant TMDC semiconductor. The monolayer MoTe_2_ possesses a direct bandgap of about 1.1 eV [44], and the bulk form exhibits an indirect bandgap of about 0.88 eV [45]. Such a small bandgap enables a large number of photogenerated carriers in the material under the pump of the commercially available 800 nm laser (photon energy of 1.55 eV). Moreover, the carrier mobility of MoTe_2_ can reach 8.5 cm^2^/V/s at the thickness of 10 nm [46]. These outstanding characteristics promote the application of MoTe_2_ in photodetectors [47], phototransistors [48], field-effect transistors [49], and sensors [50]. However, as a prominent TMDC semiconductor, the application of MoTe_2_ in the ultrafast THz switch has not been reported.

In this paper, we report an ultrafast switchable transmission amplitude modulation as well as the slow light behavior by integrating the 10-nm thick 2H-type MoTe_2_ film with the electromagnetically induced transparency (EIT) metasurface, for the first time. Based on the homemade optical pump and THz probe (OPTP) spectroscopy system, the EIT amplitude modulation depth reaches 77% under the pump fluence of 3200 μJ/cm^2^ and shows a trend of quenching with higher pump energy. Moreover, a group delay variation up to 4.6 ps is measured to characterize the tunable slow light performance. It is demonstrated that the whole switch cycle from the ‘’off’’ to ‘’on’’ state of the EIT resonance can be completed on a timescale of the sub-nanosecond, providing a useful option for the ultrafast switchable THz metadevice that may be employed in the upcoming 6G communication.

## 2. Materials and Methods

### 2.1. MoTe_2_-Covered EIT Metasurface

**Figure 1:**
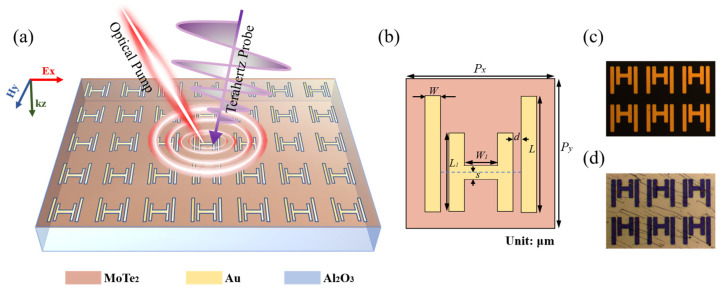
Schematic and working principle of the designed switchable EIT metasurface covered by the 10-nm thick MoTe_2_ multilayer. (**a**) An artistic illustration of the hybrid metasurface under illumination of the 800 nm femtosecond laser and THz probe pulse; (**b**) the geometrical configuration of one proposed unit cell with the following structure parameters: *Px* = 56 μm, *Py* = 58.8 μm, *W* = 5.6 μm, *L* = 42 μm, *W*_1_ = 12.6 μm, *L*_1_ = 28 μm, *d* = 3.5 μm, and *s* = 2.8 μm. (**c**,**d**) Optical microscope images of the fabricated metasurface, without MoTe_2_ and with 10-nm thick MoTe_2_ coating, respectively.

Figure 1a shows the schematic illustration of the switchable EIT metasurface device. Specifically, functional meta-atoms composed of gold H-shaped cut wires (HW) and parallel cut wires (PW) are periodically arranged to realize the EIT resonance of the incident THz waves. The detailed geometric configuration of each meta-atom is presented in Figure 1b. The metasurface was fabricated on the sapphire substrate using the conventional photolithography technology, with its optical microscopic image shown in Figure 1c. Then, the 10-nm thick 2H-type MoTe_2_ film produced by the chemical vapor deposition was transferred onto the entire metasurface via the wet-transfer method [51], to complete the fabrication of the device. The surface morphology of the MoTe_2_-coated metasurface can be clearly observed in Figure 1d. When an external 800 nm femtosecond laser pulse is irradiated on the MoTe_2_ film, a large number of electrons can be excited to affect the coupling of the bright element HW and dark element PW, leading to the quenching of the EIT resonance. Immediately after that, the free electrons will recombine with the holes, and the EIT resonance will recover. The excitation and relaxation of such photogenerated carriers can be accomplished on the timescale of sub-nanosecond, enabling the ultrafast switching of the EIT resonant states. Generally, the external laser pulse is denoted as the optical pump, and the incident THz wave is denoted as the THz probe [32,52,53].

### 2.2. Optical Pump and Terahertz Probe (OPTP) Measurement

**Figure 2:**
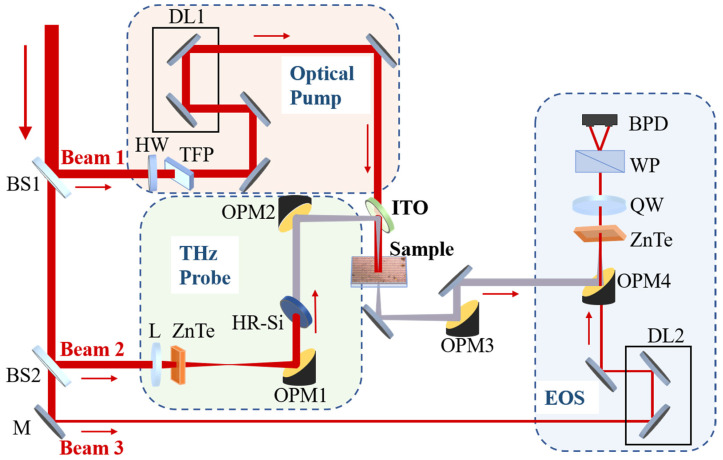
Schematic representation of the experimental setup. BS1 and BS2, beam splitters; M, mirror; L, lens; ZnTe, zinc telluride electro-optic crystal; OPM1-4, off-axis parabolic mirrors; HR-Si, high-resistivity silicon plate; ITO, indium tin oxide transparent conductive film glass; HW, half-wave plate; TFP, thin film polarizer; DL1 and DL2, delay lines; QW, quarter-wave plate; WP, Wollaston prism; BPD, balanced photodiodes.

To characterize the ultrafast switchable EIT resonance of the device, we have built an optical pump and THz probe (OPTP) spectroscopy system, as illustrated in Figure 2. A Ti: sapphire amplifier of Spectra-Physics was adopted as the laser source, with parameters of the 800 nm central wavelength, 100 fs pulse duration, 5 mJ pulse energy and 1 kHz repetition rate. The laser pulse was split into three beams for the optical pump (Beam 1), THz generation (Beam 2), and electro-optic sampling (EOS) (Beam 3). In the light path of optical pump, Beam 1 firstly passed through a half-wave plate (HW) and a thin film polarizer (TFP). By rotating the HW, the intensity of the laser transmitted from the TFP can be adjusted. Then, after passing the delay line 1 (DL1) and penetrating the indium tin oxide transparent conductive film glass (ITO), the optical pump irradiated onto the surface of the hybrid metasurface. In another light path, Beam 2 was focused by a lens (focal length of 30 cm) and then acted on the zinc telluride electro-optic crystal (ZnTe) to generate the THz emission via optical rectification. The emitted THz wave was collected by a pair of off-axis parabolic mirrors (OMP1 and OMP2), and the high-resistivity silicon plate (HR-Si) between the OMPs was used to block the 800 nm laser and transmit the THz emission. It is worth noting that the ITO can transmit the infrared wave and reflect the THz wave. Therefore, the optical pump from Beam 1 and the THz probe from Beam 2 can act on the hybrid metasurface jointly. To ensure a fully adequate optical excitation, the spot size of the optical pump beam on the sample was arranged to be larger than the THz beam. Moreover, by moving the translation stage of DL1, the optical path difference between the optical pump and THz probe can be changed to adjust the pump-probe delay, which is the key point for the characterization of the ultrafast dynamics. Then, the modulated THz waveform can be recorded by the EOS measurement with Beam 3, and the corresponding THz spectrum can be obtained by the standard Fourier transformation.

## 3. Results

### 3.1. Simulation

In order to clarify the mechanism behind the modulation of MoTe_2_-film-covered EIT metasurface, we have performed the numerical simulation by using the CST Microwave Studio Software in the time domain. The fabricated metasurface has a size of 10 mm × 10 mm. Here, we only need to simulate a unit cell of 56 μm × 58.8 μm size with the “Periodic” boundary conditions imposed in the *x*- and *y*-directions. The THz wave is set as a vertically incident plane wave polarized along the *x* direction. A probe is placed at the bottom of the sapphire substrate to record the transmitted THz waveform.

**Figure 3:**
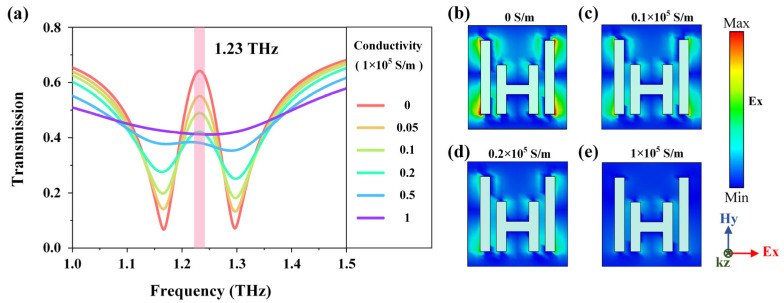
Simulated THz modulations of the hybrid EIT metasurface covered by the 10-nm thick MoTe_2_ film. (**a**) THz transmission spectra with various conductivity of the MoTe_2_ film from 0 S/m to 1 × 10^5^ S/m. (**b**–**e**) Near E-field distributions above the metasurfaces at frequency of 1.23 THz within a unit cell.

As mentioned above, photogenerated carriers can be excited by the external laser pulse and will change the conductivity of the MoTe_2_ film. Without laser pumping, we consider that the initial conductivity of the MoTe_2_ film approximates to 0. For incident THz waves polarized along the *x*-direction, near-field coupling occurs between the H-shaped cut wire and parallel cut wires, leading to the high transmittance at 1.23 THz, as shown by the red line in Figure 3a. The corresponding near-field distribution is presented in Figure 3b, from which it can be observed that at the EIT peak frequency, the electric field of the H-shaped cut wire is suppressed, and the electromagnetic energy is transferred to the parallel cut wires. As we increase the conductivity of MoTe_2_ film, the transmission amplitude of the EIT window gradually decreases, and finally, the EIT phenomenon disappears at the conductivity of 1 × 10^5^ S/m (Figure 3a). Meanwhile, the near-field coupling gradually weakens so that less energy is transferred to the parallel cut wires, which is manifested as the large reduction of the electric field intensity above the metasurface, as shown in Figure 3c–e. These simulations help us to design and carry out the following experiments.

### 3.2. Experimental Results

#### 3.2.1. Pump-Fluence Controlled THz Transmission and Slow Light Behavior

**Figure 4:**
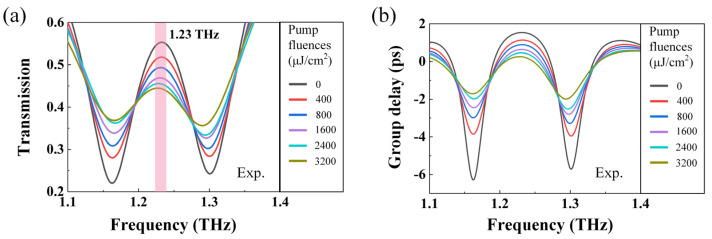
Pump fluence-dependent EIT response of the MoTe_2_-coated metasurface. (**a**) Measured THz transmission spectra for different pump fluence from 0 to 3200 μJ/cm^2^. (**b**) Measured group delays for different pump fluence from 0 to 3200 μJ/cm^2^.

Based on the homemade OPTP system, we have investigated the optical performance of the fabricated MoTe_2_-coated metasurface. Firstly, the THz transmission spectra under various pump fluences were measured and compared to validate the feasibility of active modulation, as presented in Figure 4a. It can be found that the EIT window is suppressed significantly with increasing pump fluence, which is consistent with the simulation of the photoconductivity-dependent EIT resonance. The deviation of the transmission amplitude in the measurement might be introduced by the dielectric loss as well as the slight differences of the parameters and boundary conditions between the simulation model and the sample. To quantitatively evaluate the modulation effect, the modulation depth defined by the expression $MD_{EIT}=\left(T_{0}-T_{pump}\right)/T_{0}\times 100\%$ is adopted [54]. In the expression, $T_{0}$ is the EIT amplitude without the pump and $T_{pump}$ is the EIT amplitude under the optical pump. The EIT amplitude is defined as the transmission amplitude difference between the peak (at 1.23 THz) and the valley (at 1.16 THz) of the EIT resonance [55].

When the pump fluence reaches 3200 μJ/cm^2^, the EIT modulation depth is calculated to be 77%, which is similar to the simulated modulation depth with the MoTe_2_ conductivity of 2 × 10^4^ S/m. Generally, photoconductivity is proportional to the product of the carrier mobility μ and the carrier density n, with the expression of $\sigma =\mu_{e}n_{e}e+\mu_{h}n_{h}q$. Based on the electron mobility of 8.5 cm^2^/V/s in 10-nm thick MoTe_2_ given by Ref [46], the carrier density under the pump fluence of 3200 μJ/cm^2^ is estimated to be 1.47 × 10^26^ m^–3^, with the photoconductivity induced by the hole being ignored due to the fact that $\mu_{e}\gg \mu_{h}$. Here, 3200 μJ/cm^2^ is the maximum pump fluence we can obtain in the experiment. If the pump laser energy can be further enhanced, it would be expected to achieve a larger modulation depth.

In addition to the pump fluence-dependent transmission spectra, the MoTe_2_-coated metasurface also exhibits an actively controlled slow light behavior [56]. To characterize the slow light performance, the group delay defined as $\Delta t_{g}\left(\omega \right)=-d\phi /d\omega$ is employed, where ϕ is the relative phase of transmitted THz wave compared with that from the pure sapphire substrate, and ω is the angular frequency of the THz wave. The spectra of $\Delta t_{g}$ at different pump fluence is plotted in Figure 4b. Noted that the group delay reaches –6.3 ps at the EIT dip (1.16 THz) with no photo-injection, and increases to –1.7 ps at the maximum pump fluence. Such a high modulation depth of 4.6 ps can provide a wide manipulation range for light–matter interaction.

#### 3.2.2. Ultrafast Dynamics of the Switching

In the last section, we have investigated the effect of pump fluence on THz transmission amplitude and group delay. It should be pointed out that the measurement was performed with a pump–probe delay of 0 ps to ensure the maximum modulation depth. The pump–probe delay is a variable that is used to represent the time difference to reach the metasurface between the THz pulse and the pump laser pulse. When the delay equals to 0, it implies the highest concentration of the photogenerated carriers when the THz probe interacts with the metasurface. The negative sign of the pump–probe delay means that the THz pulse reaches the metasurface earlier than the pump laser, whereas the positive sign represents the opposite.

**Figure 5:**
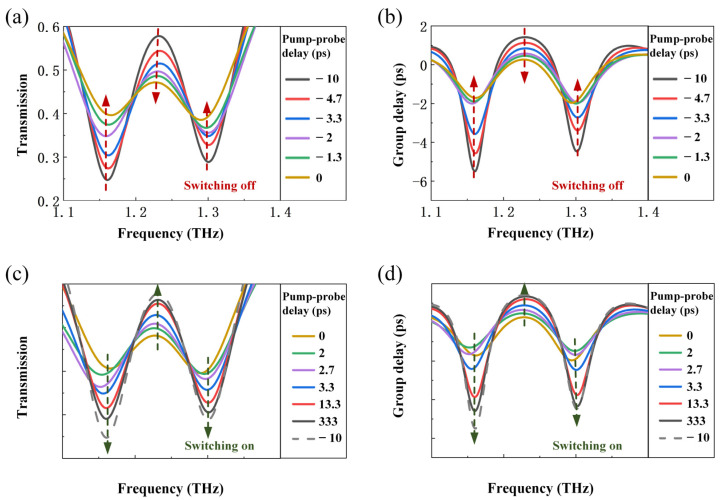
Ultrafast switching of the EIT resonance under pump fluence of 3200 μJ/cm^2^. (**a**,**b**) The switching-off process of transmission spectra and group delay spectra as a function of pump–probe delay, respectively. (**c**,**d**) The switching-on process of transmission spectra and group delay spectra at various pump-probe delays, respectively.

Next, we will show the THz transmission spectra and group delay spectra at different pump-probe delays under the fixed pump fluence of 3200 μJ/cm^2^, to manifest the ultrafast switch of the EIT response. Figure 5a,b show the switching-off process of the EIT resonance, which corresponds to the excitation of photogenerated carriers. Herein, the pump–probe delay starts from –10 ps. In that case, the pump laser pulse incident on the metasurface just met the ‘tail’ of the THz probe pulse, and the induced carriers do not affect the EIT resonance. As the pump–probe delay gradually increases to 0, the pump pulse starts to catch up with the main peak of the THz pulse, and more photogenerated carriers are concentrated to diminish the EIT response. After that, the switching-on processes are plotted in Figure 5c,d, with the EIT resonance displaying an explicit trend of recovery. It can be observed that the switching-on process of the EIT takes longer time than the switching-off process, depending on the excitation and relaxation of the carriers. For the 10-nm thick MoTe_2_ film in our experiments, photogenerated carriers can be excited in a few picoseconds, but take hundreds of picoseconds for relaxation.

Finally, to better analyze the performance of the MoTe_2_-covered ultrafast all-optical tuning metasurface, the parameters of relevant research based on the typical semiconductors [33,57] and another kind of TMDC [55] are listed in Table 1. All of the hybrid metasurfaces listed are in the EIT resonance mode and are pumped by the laser of the 800 nm wavelength. By comparison, it is found that the hybrid metasurface with 10-nm thick MoTe_2_ film possesses the shorter switching time than that with the 500-nm-thick Si film [33], and its pump threshold is of the same order of magnitude as that with the 200-nm-thick Ge film [57]. Moreover, compared with the 40-nm thick WSe_2_ [55], which also belongs to the TMDCs, the 10-nm thick MoTe_2_ can achieve a greater modulation depth within the designed metasurface. Although the pump threshold of MoTe_2_ is not dominant, it is important to note that the film thickness we applied is the thinnest. As silicon-based devices approach the limits of Moore’s Law, processes below 14 nm are increasingly challenging. The quasi-two-dimensional MoTe_2_ with the thickness of 10 nm in our work has demonstrated the potential to surpass the silicon-based device in response speed, providing a promising candidate for the on-chip device. Moreover, the comparison of the simulated photoconductivity implies that 10-nm-thick MoTe_2_ might possess an ultrahigh optical conductivity, demonstrating competitiveness in the promising application of photoelectric devices.

## 4. Conclusions and Perspective

In summary, we have demonstrated an all-optically controlled ultrafast switching of the THz transmission and slow light behavior by integrating the 2H-type MoTe_2_ thin film with the EIT metasurface for the first time. The entire switching cycle can be completed in sub-nanoseconds, originating from the dynamic properties of the photocarriers in the MoTe_2_ film. Based on the experimental system of OPTP, the THz transmission amplitude modulation up to 77% is achieved under a pump fluence of 3200 μJ/cm^2^. In addition, a group delay variation as high as 4.6 ps is observed, indicating the slow light effect introduced by the fast change of refractive index on the resonant frequency of the designed EIT metasurface.

Our work provides a new candidate for on-chip devices on 6G communication, as well as an extraordinary slow light device to be applied in nonlinear optics, optical storage, and so on. The indirect band gap of MoTe_2_ implies a possible application for near-infrared fiber communication located at the O-band (1260 nm–1360 nm) [58], enabling a hybrid control and ultrafast trigger of the infrared and THz waves. Furthermore, recent advances of the optically controlled THz metasurface have experimentally demonstrated a calibration-free sensor for achieving high-precision biosensing detection [31]. Since the biological interest concerns the full spectral range from THz wave to the mid-infrared (MIR) [59,60], the resonant frequency of the matasurface can also be extended to the MIR for more practical applications.

## Figures and Tables

**Table 1:** Comparison of the ultrafast all-optical tuning metasurfaces.

Material	Thickness (nm)	Switching Time (ps)	Pump Threshold (μJ/cm^2^)	Simulated Photoconductivity (S/m)	Modulation Depth
[33] Si	500	780 (half-recovery state)	200	600	100%
[57] Ge	200	15	2200	1000	100%
[55] WSe_2_	40	8	800	4800	43%
MoTe_2_	10	<300 (half-recovery state)	3200	>2 × 10^4^	77%

## Data Availability

Data underlying the results presented in this paper are not publicly available at this time but may be obtained from the authors upon reasonable request.
